# Invasive Procedures to the Neck and Chest and Hemidiaphragmatic Paralysis: Are They Always Causally Associated?

**DOI:** 10.7759/cureus.74656

**Published:** 2024-11-28

**Authors:** Amanda Darmanin, Yanika Gatt, Mark Miruzzi, Martin V Balzan

**Affiliations:** 1 General Medicine, Mater Dei Hospital, Msida, MLT; 2 Respiratory Medicine, Mater Dei Hospital, Msida, MLT; 3 Medicine, Mater Dei Hospital, Msida, MLT

**Keywords:** central vein cannulation, cervical root compression, diaphragm, hemidiaphragmatic paralysis, implantable cardioverter defibrillator, phrenic nerve injury, thoracic procedures

## Abstract

Three cases of hemidiaphragmatic paralysis are reported. One case was associated with an interscalene brachial plexus block, another with the insertion of an implantable cardioverter defibrillator, and a third case had undergone a coronary artery bypass grafting operation. In only one of these cases, there was a causal association, while in the other two, it was determined that the paralysis was coincidental. A change in the diaphragmatic position on the chest X-ray needs to be demonstrated, preferably also confirmed by ultrasound or a fluoroscopic sniff test, to show that the paralysis was a clear and immediate consequence of the procedure.

One case described was most likely related to cervical nerve C3-C5 root compression confirmed on a magnetic resonance imaging (MRI) scan and not due to the procedure. This case shows that cervical root compression at C3-C5 by disk prolapse should also be considered. This was also suspected in another case; however, an MRI could not be performed after discussing the risks with the patient.

When the paralysis has no temporal relationship with the procedure, primary or secondary intrathoracic malignancy and concurrent neurological disease must also be excluded by computed tomography of the thorax. The surgical and interventional causes of phrenic nerve injury and the relative evidence base are discussed.

## Introduction

The diaphragm is an essential respiratory muscle, and its weakness can impair respiratory function. Unilateral diaphragmatic weakness occurs when it is compromised due to damage to the phrenic nerve, often as a consequence of an underlying medical condition or an invasive intervention along its course. The palsy may be reversible, partially reversible, or permanent. Transient lesions may follow local anesthesia or hypothermic injury, while permanent lesions are usually caused when the nerve is either transected surgically or infiltrated by a tumor [1,2].

Iatrogenic causes can occur along any part of the long anatomic course of the phrenic nerve. Damage may occur following head and neck procedures [3], upper gastrointestinal surgery [4], and thoracic surgery [5], including coronary artery bypass grafting (CABG). Other traumatic causes include injuries secondary to gunshot wounds or deceleration accidents due to motor vehicle accidents or falls from height [6]. Hemidiaphragmatic paralysis (HDP) may also occur due to compression of the phrenic nerve due to space-occupying lesions (both primary and secondary tumors) [7] and cervical spondylosis [8], as well as neurological conditions such as multiple sclerosis [9], Guillain-Barré syndrome [10], and myasthenia gravis [11].

Sometimes, patients are labeled as having an iatrogenic cause of unilateral diaphragmatic paralysis, but with a more detailed history and chart/image review, this can be due to other unrelated causes. Three cases of unilateral diaphragmatic paralysis associated with an invasive intervention are presented here; however, two of which turned out to be unrelated to the procedure performed.

## Case presentation

Case 1

**Figure 1:**
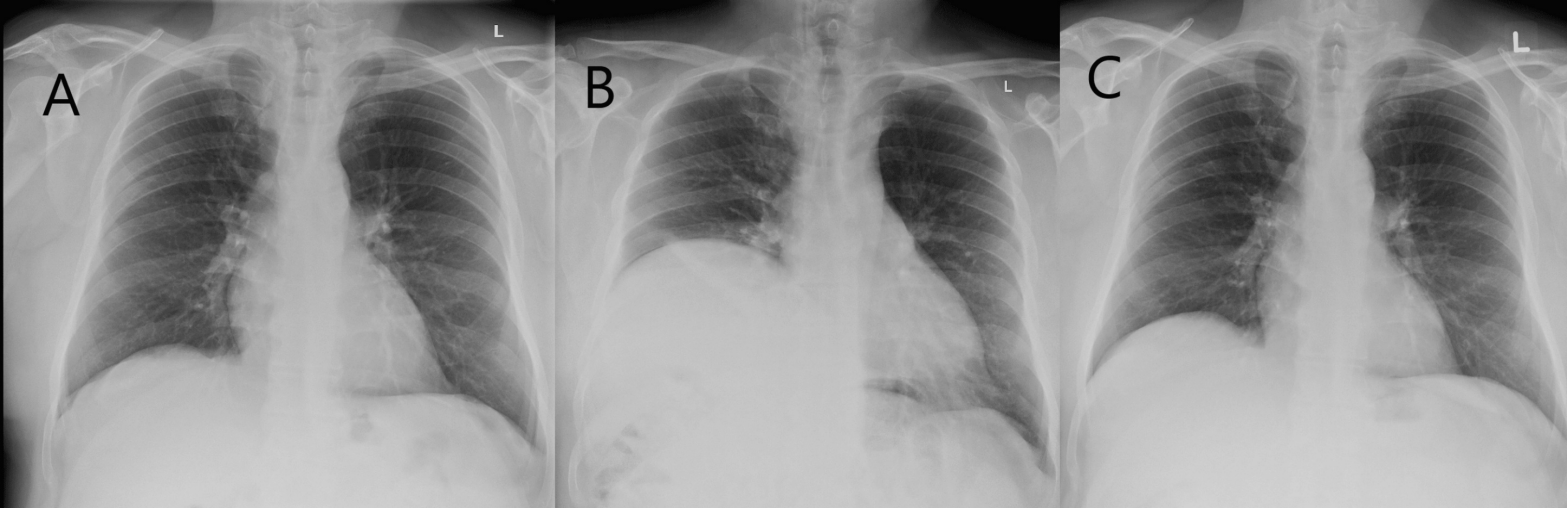
CXRs before and after the procedure using interscalene local anesthesia. (A) Normal preoperative CXR. (B) Day 1 postoperative, showing grossly elevated hemidiaphragm. (C) Day 3 postoperative, showing the paralysis having been resolved and the hemidiaphragm returned to a normal position CXR: chest X-ray

This case is a 44-year-old male patient known to suffer from adult-onset asthma well controlled with budesonide 200 µg inhaler twice daily, a thalassemia trait, and anxiety treated with paroxetine 20 mg daily. He had no known drug allergies. He underwent a right arthroscopic rotator cuff tear repair of his right shoulder under the care of an orthopedic surgeon. Before surgery, the patient was given a right interscalene brachial plexus block in the neck, using 30 mL of bupivacaine and 8 mg of dexamethasone under ultrasound guidance. Pulse, respiratory rate, and blood pressure were within normal limits during the procedure. Preoperative chest X-ray (CXR) was normal (Figure 1A).

The rotator cuff repair procedure was performed successfully. Postoperatively, the patient was deemed fit to be discharged home; however, in the evening, while lying down, the patient developed dyspnea, which was aggravated while lying on the right side. The patient also complained of mild chest discomfort. On examination, he was noted to be tachypneic at 24 breaths/minute but was able to complete sentences. While breath sounds were noted to be fainter on the right, there were no added sounds or wheezing.

CXR (Figure 1B) showed an apparent elevation of the right hemidiaphragm (>5 cm above the left diaphragm). However, an ultrasound examination was not performed by the caring physician at the time. Given the clear change in the position of the diaphragm, D-dimer and computed tomography (CT) pulmonary angiography were not performed. He was kept in the hospital for observation and oxygen therapy. His dyspnea quickly improved over the next two days, while the CXR (Figure 1C) showed that the diaphragm had returned to its original position. At this point, it was considered that the temporary phrenic nerve palsy was more likely to be due to diffusion of local anesthetic rather than direct injury to the phrenic nerve.

Case 2

A 49-year-old male patient underwent the insertion of a dual-chamber implantable cardioverter defibrillator (ICD) given recurrent episodes of wide complex ventricular tachycardia (VT) at 200 beats/minute. The patient's past medical history included episodes of depression and right inguinal hernia surgical repair. An echocardiogram showed a left ventricular ejection fraction of 55%, with no evidence of valvular or structural heart disease (Video 1).

**Video 1:** Echocardiogram after ICD insertion The LV dimensions are normal, with global LV systolic function estimated to be low normal. The ejection fraction is estimated at 55% using the Simpson biplane method. No significant regional wall motion abnormalities were observed. The LA size is normal, with an end-systolic volume of 51 mL, and the interatrial septum is intact. The tricuspid aortic valve is thin and opens well, with no aortic regurgitation detected. The mitral valve leaflets, also thin and mobile, open well but show trace mitral regurgitation. The RV size and RV systolic function are normal, with an end-diastolic area of 17 cm^2^ and an end-systolic area of 8 cm^2^. The fractional area change is over 50%. The right atrial size is normal, with an end-systolic area of 11 cm^2^. The pulmonary valve exhibits trace pulmonary regurgitation. The tricuspid valve is normal, showing trace tricuspid regurgitation with a maximum gradient of 23 mmHg. The inferior vena cava is not dilated and collapses well on inspiration. There is no pericardial effusion. In conclusion, the LV dimensions are normal, with low normal global LV systolic function. No significant valvular pathologies were noted. The bright spots on the right side are typical of the atrial and ventricular leads of the ICD. (Press full-screen bottom right, right click on the screen to loop.) ICD: implantable cardioverter defibrillator; LV: left ventricular; LA: left atrial; RV: right ventricular

A dual-chamber magnetic resonance imaging (MRI)-conditional ICD was inserted via the left subclavian vein (access via the left pectoral subcutaneous region) under local anesthesia at the area of insertion and strict aseptic conditions. The right ventricular defibrillation lead was positioned in the right ventricular septum. An active fixation right atrial lead was positioned in the right atrial appendage. The base rate was set at 60 beats/minute. The patient was advised not to drive. Repeated device checks were performed to ensure that further episodes of VT were prevented.

**Figure 2:**
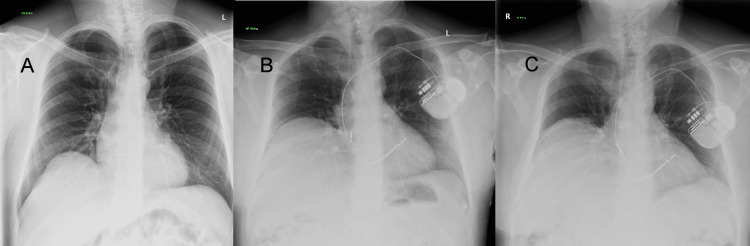
CXR showing raised hemidiaphragm. (A) Four years prior to procedure. (B) Day 1 postoperatively. (C) Day 7 postoperatively Caring physicians compared the postprocedure (B,C) CXRs with another taken 4 years before the episode (A), which had already revealed a lesser degree of preexisting right hemidiaphragm elevation. However, it appeared to be greatly accentuated postoperatively

**Figure 3:**
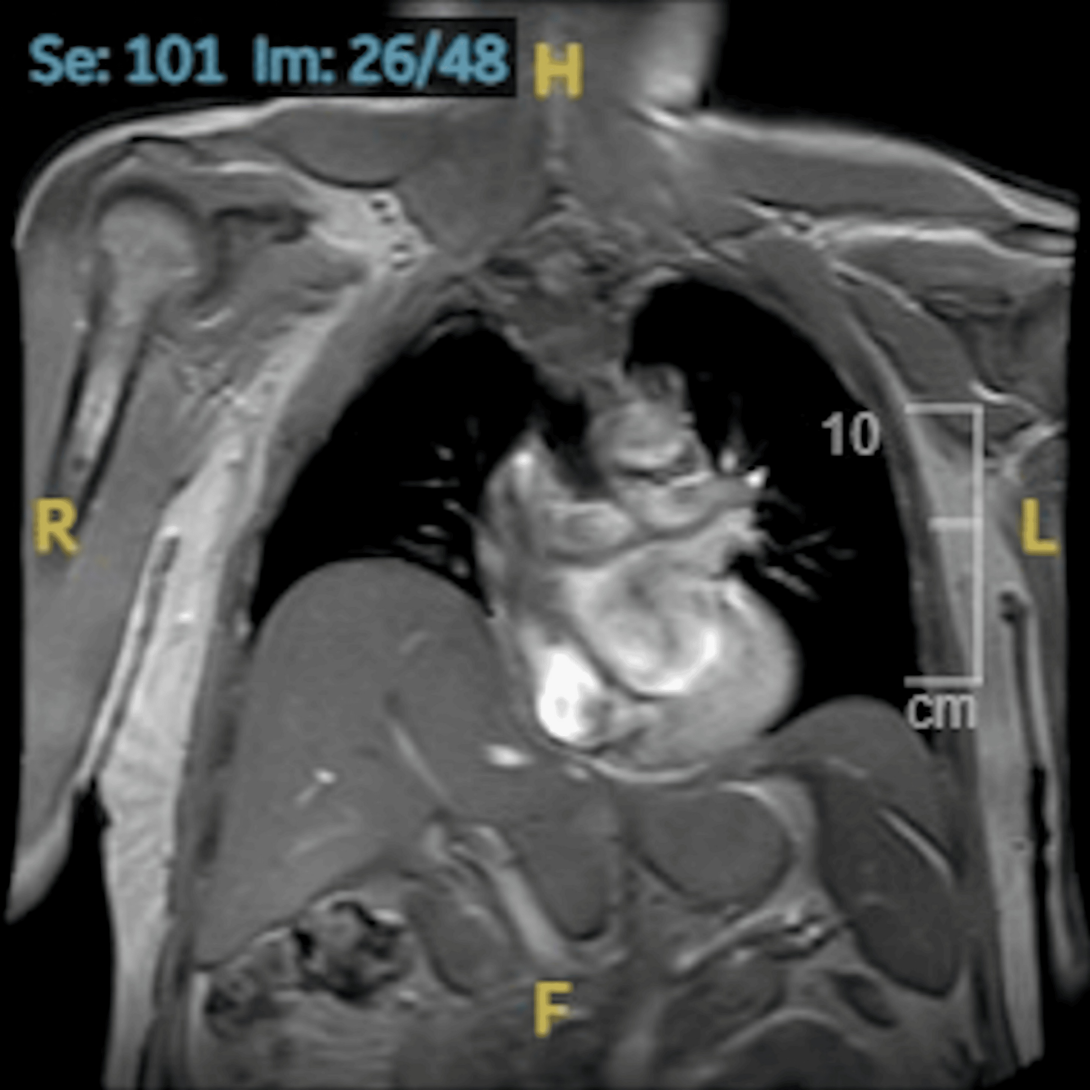
Cardiac MRI three days prior to ICD insertion The MRI image taken three days before ICD insertion shows a raised hemidiaphragm, looking very similar to the CXR image postoperative. Please note that the MRI scan was taken supine, while CXR was taken erect, which is a limitation of the comparison. However, the shape of the diaphragm and the vertical distance between the left and right diaphragm are quite similar ICD: implantable cardioverter defibrillator; MRI: magnetic resonance imaging; CXR: chest X-rays

Caring physicians compared the postprocedure CXR (Figures 2B, 2C) with another taken four years before the episode (Figure 2A), which had already revealed a degree of preexisting right hemidiaphragm elevation. However, it appeared to be accentuated postoperatively. A second look at the cardiac MRI taken three days before the procedure (Figure 3) showed that the picture was similar to the CXR taken postoperatively. As cannulation of the subclavian vein had been on the left while the right diaphragm was raised, it was concluded that the two pathologies were completely unrelated. Video 1 shows an echocardiogram post-ICD insertion.

The authors referred the patient for further investigation. Paralysis of the right hemidiaphragm was confirmed on ultrasound. The patient had no history of trauma to the chest, no documented episodes of cardiac resuscitation, or indeed of any neck, thoracic, or abdominal surgery. He did not complain of any symptoms of cervical nerve root compression. Additionally, there were no sensory deficits or any other neurological signs during the physical examination. CT thorax was not performed, given the long-standing nature of the paralysis. An MRI of the cervical spine was considered; however, given the risks associated with the presence of the ICD device and the absence of symptoms, it was not performed after discussion with the patient. For this reason, no clear cause could be determined.

Case 3

A 77-year-old male patient, known to suffer from hypertension, dyslipidemia, and ischemic heart disease, had undergone a CABG x2 procedure in 2007 because of significant stenosis of the left anterior descending artery and right coronary artery. The left internal mammary artery had been anastomosed to the left anterior descending artery, while the greater saphenous vein was anastomosed to the posterior descending artery. No postoperative complications were reported.

**Figure 4:**
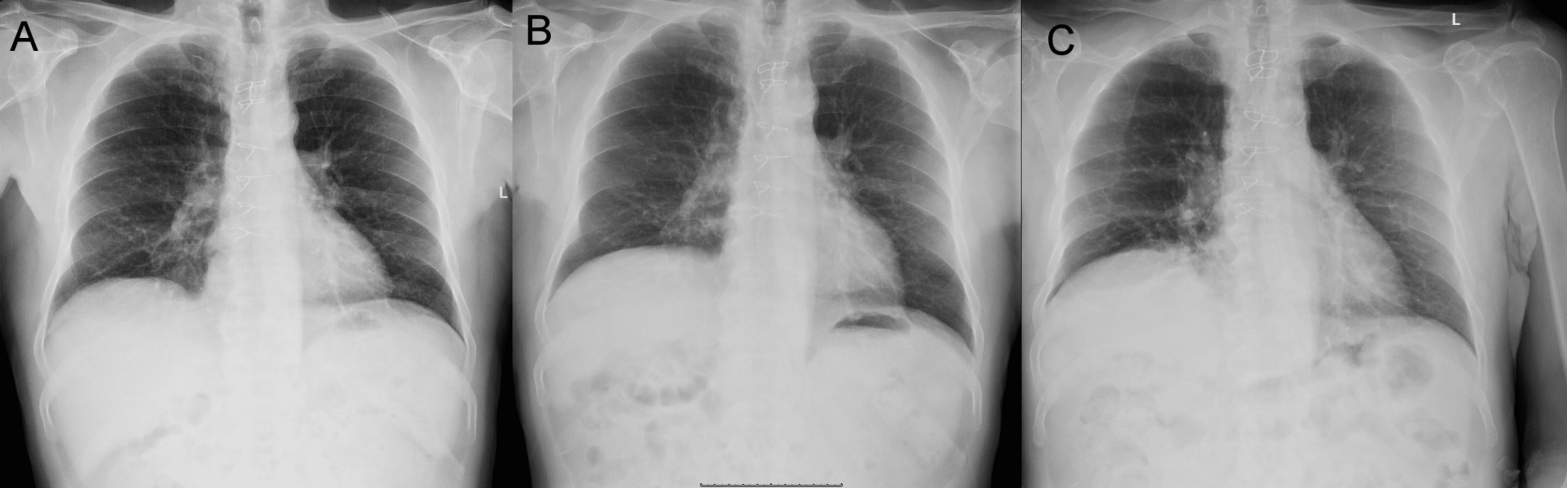
CXR time series after CABG: (A) 2007, (B) 2012, and (C) 2021 The postoperative CXR in 2007 (A) showed no evidence of hemi-diaphragmatic elevation, while the CXR performed in 2012 (B) showed an intermediate position. The raised hemidiaphragm was still present in 2021 (C) CXR: chest X-ray; CABG: coronary artery bypass grafting

A CXR performed in 2021, apart from the sternal wires, revealed an elevated right hemidiaphragm as seen in Figure 4C. However, the postoperative CXR in 2007 (Figure 4A) showed no evidence of hemidiaphragmatic elevation, while the CXR performed in 2012 (Figure 4B) showed an intermediate position. The patient denied any accidents/falls and had not undergone any thoracic surgical procedures in the interim, making the etiology of the diaphragmatic elevation unclear.

**Figure 5:**
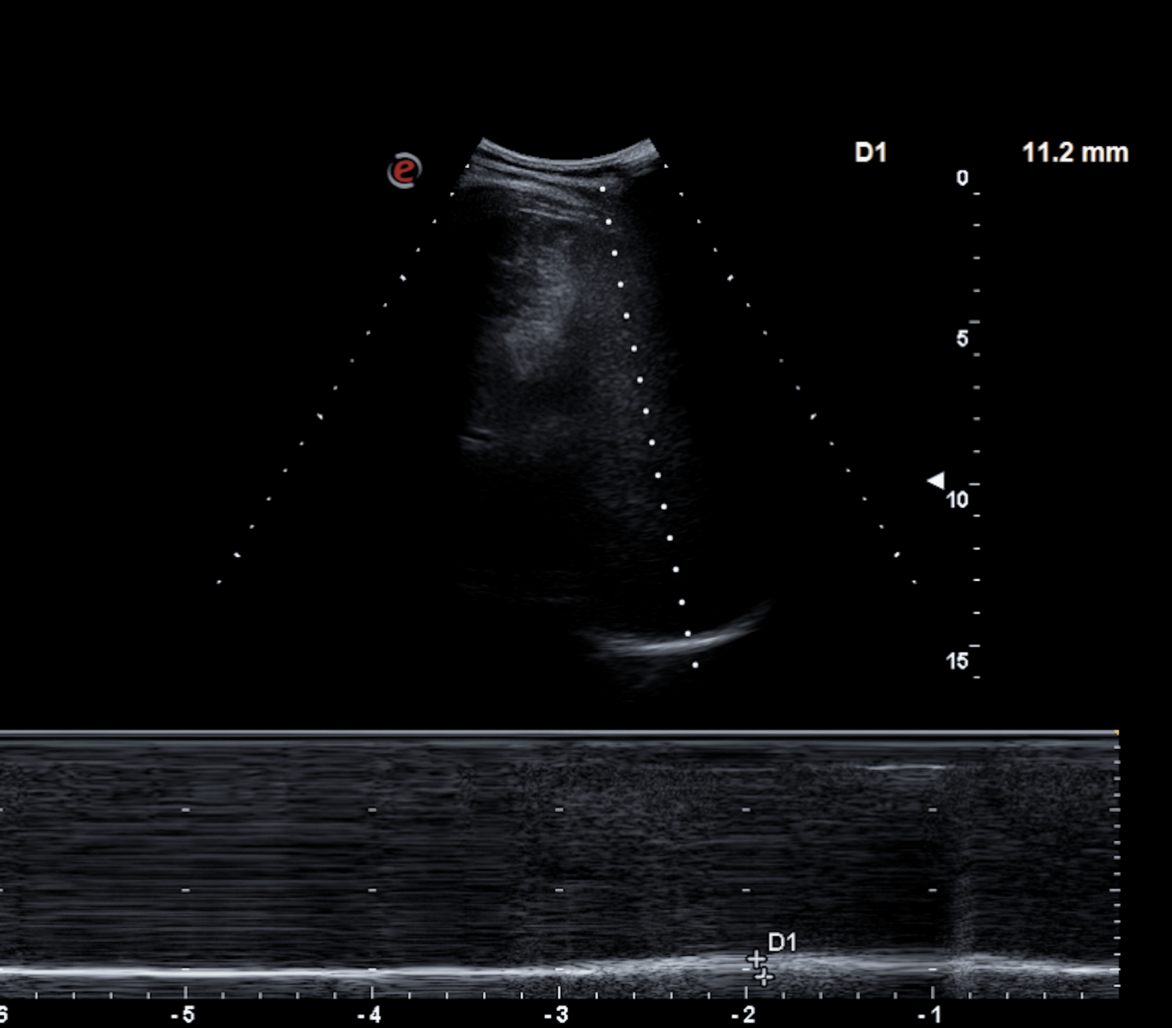
M-mode thoracic ultrasound showing reduced right hemidiaphragmatic excursion (<1 cm), indicative of partial right hemidiaphragmatic paralysis The two crosses in the bottom line labeled as D1 are very close indicating a distance of <1 cm

During this period, the patient underwent a right inguinal hernia repair and right carpal tunnel decompression. Figure 5 shows a point-of-care ultrasound image taken in 2024, which revealed persistent suboptimal movement of the right hemidiaphragm on inspiration and expiration, indicating a persistent degree of diaphragmatic paralysis.

**Figure 6:**
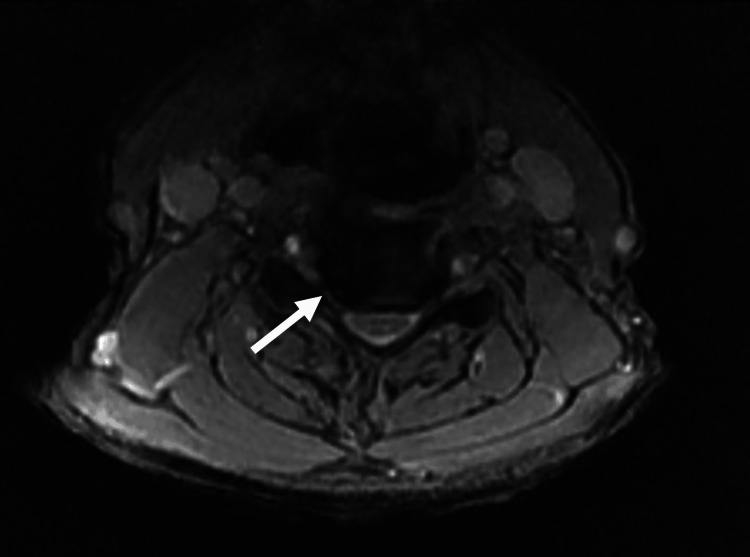
An axial T2W image at the level of C4-C5 showing a right-sided neuroforaminal stenosis (arrow) T2W: T2-weighted

**Figure 7:**
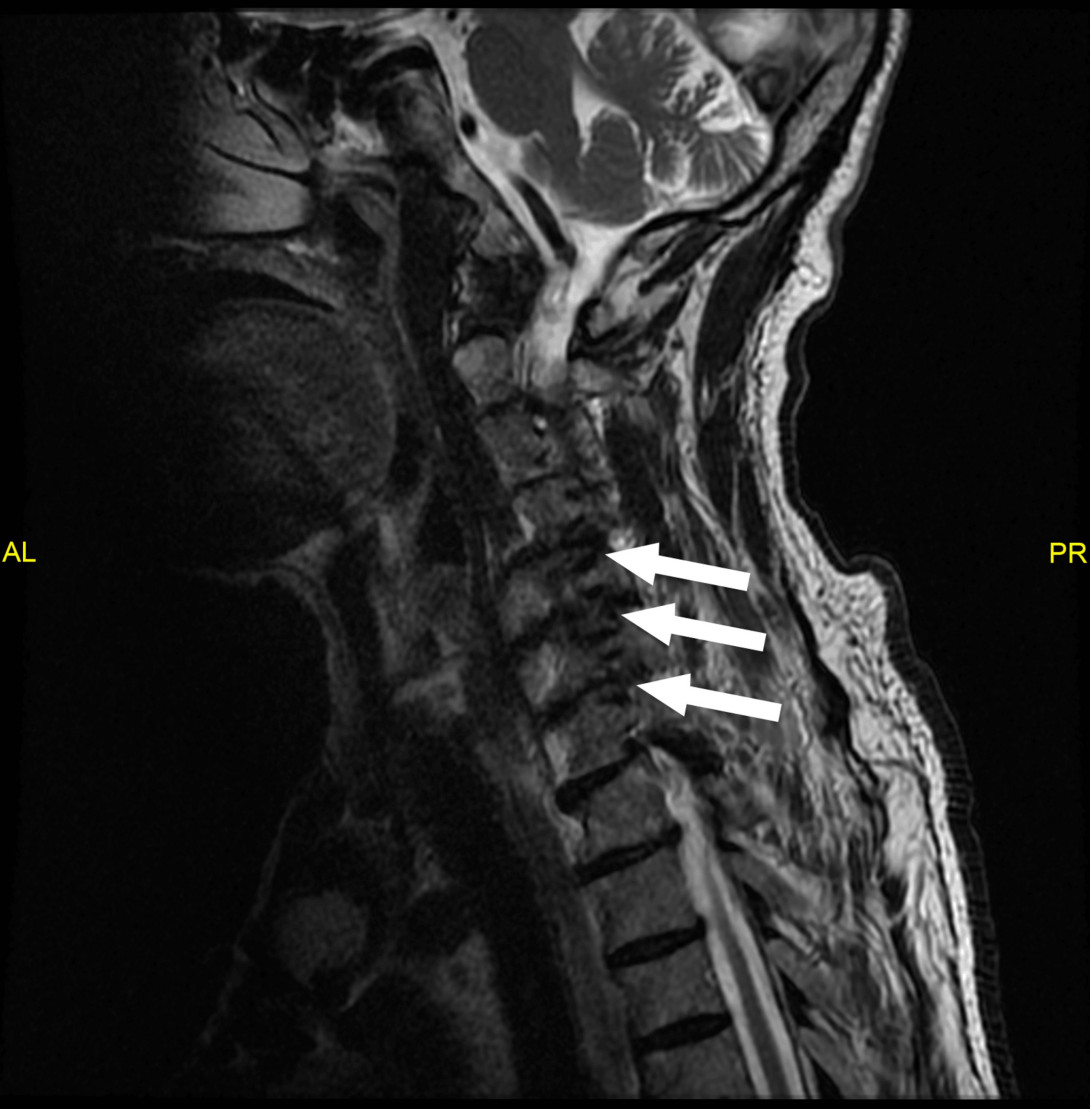
Sagittal T2W image through the cervical spine showing the stenosed C4-C7 neuroforamina (arrows) T2W: T2-weighted

The patient complained of shortness of breath on exercise. He also complained of bilateral paresthesia and pain in both arms. An MRI of the cervical spine from C3-C5 is shown (Figures 6, 7). The progressive compression of the cervical nerve roots was considered the most likely cause of the paralysis. A CT thorax was not performed because of the long-term nature of the event.

## Discussion

These three cases serve to highlight the fact that HDP should be one of the important differential diagnoses in patients presenting with respiratory symptoms after surgical procedures in the neck and chest [12]. However, in two of the cases reported here, the intervention/surgery was not related to the onset of the phrenic nerve palsy and was only coincidental. In such cases, other causes, such as thoracic malignancy and neurological disease, particularly cervical root compression at C3-C5, should be considered [8].

The diaphragm is the most important respiratory muscle and is vital for ventilation as it pushes the viscera downward and enhances rib movement during respiration [13]. Diaphragmatic paralysis can be unilateral or bilateral, the former being much more common [12]. In the presence of diaphragm weakness, there is a decreased or absent contraction of this muscle, leading to less caudal displacement. This leads to less expansion of the thoracic cavity and, therefore, less air movement into the lung, causing hypoventilation. Where paralysis of both hemidiaphragms occurs, it may lead to respiratory failure [14].

However, unilateral paralysis is asymptomatic in most patients, as the contralateral diaphragm and external intercostal muscles compensate for it. Paralysis of the diaphragm can result either from the intrinsic weakness of the diaphragm muscles or, more commonly, from damage to the phrenic nerves. In phrenic nerve injury, depending on the cause, the palsy may be temporary or permanent [14].

The phrenic nerve originates from the C3, C4, and C5 nerve roots. It arises in the neck, at the upper border of the scalene muscle, which lies close to the brachial plexus. From there, it descends to the thorax; the left and right phrenic nerves lead different paths. The left nerve runs down in front of the hilum of the left lung, crosses anterior to the aortic arch and the pericardium of the left ventricle, and then reaches the diaphragm close to the heart apex. The right side has a shorter route, as it descends anterior to the hilum of the right lung and then passes along the pericardium of the right atrium to reach the diaphragm [15]. Given the longer route of the left phrenic, left diaphragm palsy is more common [14].

Damage to the phrenic nerve may occur either during surgery or by mechanical trauma. Most cases are not symptomatic and, therefore, will not be picked up postoperatively or after the trauma and are identified incidentally on future imaging [16].

Coronary artery bypass surgery is the most common surgical cause of raised hemidiaphragm, as it may cause phrenic nerve cold injury during cold cardioplegia [5,17]. Furthermore, harvesting of internal mammary arteries can also result in mechanical injury due to the proximity of the chest wall as the phrenic nerve passes through the apex of the heart [5]. However, any other procedure along the route of the phrenic nerve can lead to phrenic nerve palsy.

Phrenic nerve injury may occur following a brachial plexus block, which is a common procedure performed for upper limb surgeries [18]. The incidence of HDP depends on the block type and the anesthetic amount given [19]. The temporary form may be as high as 92%-100% if the block is interscalene and 20 mL of local anesthetic is administered [20]. All patients show an evident decrement in forced expiratory volume in one second, forced vital capacity, and peak expiratory flow at three and eight hours after an inter scalene block [21]. On the other hand, the risk of HDP in a combination of infraclavicular block and anterior suprascapular is around 5% [19].

Robaux et al. [22] further investigated prolonged phrenic nerve paralysis after interscalene brachial plexus block with electromyography by applying phrenic nerve stimulation in the neck and then measuring conduction velocities and phrenic nerve latencies. Data showed that the right phrenic nerve was either significantly demyelinated or entirely disrupted. Moreover, patients still complained of exertional shortness of breath one year postoperatively with no functional improvement.

In another study by Kim et al. [20], the conventional volume of local anesthetic was compared to a lower experimental volume of local anesthetic at a particular position using an ultrasound-guided two-point injection technique to investigate the incidence of inter scalene brachial plexus block on HDP. Results showed that selective injection of reduced local anesthetic volume and monitoring of the spread pattern showed a lower incidence of HDP.

In case 2, HDP preceded cardiac implantable electronic device (CIED) insertion. The procedure was clearly not the cause of the nerve palsy as it was on the right side while the approach was from the left subclavian vein. López-Gil et al. [23] reported that 4 out of 626 patients experienced phrenic paralysis after CIED insertion via puncture of the subclavian vein. They suggest that this could be due to the infiltration of local anesthetic in the subclavian area in those patients with phrenic nerve course being anatomically variant. Complications may be severe but nearly always transient and can be managed conservatively. Risks may be reduced by avoiding access too medial to the subclavian vein and by limiting local anesthetic infiltration [23].

The third case of HDP was related to, but not caused by, a CABG procedure, which is known to be a cause of temporary and permanent diaphragm paralysis. The use of ice during cardiac cooling and traction of the phrenic nerve might cause temporary neuropraxia of the phrenic nerve, causing temporary diaphragm paralysis [24]. A rare complication of intrathoracic surgery is the severance of the phrenic nerve, which would result in permanent paralysis of the diaphragm [25].

Katz et al.'s study [26] noted that out of 49 patients, 13 (26%) did not completely recover their diaphragmatic function after being followed up for a mean of 32.8 months. They observed that risk factors for injury of the phrenic nerve included the length of the procedure, cold cardioplegia, topical cooling using ice slush, body temperature, number of grafts performed, presence of chronic obstructive pulmonary disease, and heart failure. The risk factors for persistent phrenic nerve injury (PPNI) following CABG are unknown. However, this study noted that patients with PPNI had a higher baseline partial pressure of carbon dioxide than patients who recovered their phrenic nerve function, suggesting that patients with some degree of ventilatory compromise have a higher risk of PPNI following CABG. The explanation for this, however, remains uncertain [26].

Mehta et al. [24] also focused on comparing the incidence of HDP after conventional CABG to off-pump CABG and the effectiveness of chest physiotherapy on diaphragmatic palsy postoperatively. The incidence of diaphragmatic palsy is exceptionally lower in adult patients since most cardiac procedures occur off-pump, followed by intensive chest physiotherapy shortly after being extubated, which helps achieve the nearly complete recovery of the diaphragmatic palsy. This causes a significant improvement in functional results after diaphragmatic palsy after cardiac surgery. The study also noted that ultrasonography is a simple yet valuable bedside means of diagnosing diaphragmatic palsy rapidly [24].

The third case also highlights that unilateral HDP is not always attributable to a surgical procedure. Fiott et al. [12] highlighted a case of respiratory failure secondary to a left HDP due to left phrenic neuropathy secondary to cervical spondylosis affecting the origin of the left phrenic nerve. In cases where no surgical procedure is responsible for the HDP, further imaging investigations are important to identify the cause.

Laghi et al. [27] describe how ultrasound is becoming the main tool to investigate the function of the diaphragm, slowly replacing the traditional "sniff test" using X-ray fluoroscopy. The limitations of ultrasound are that ultrasound imaging is "operator-dependent" and "position-dependent" with repeatability and reproducibility of 17% and 18%, respectively, and that the diaphragm may not be located in up to 10% of cases [27]. MRI is likely the preferred tool for diaphragmatic surgery [27]. A more detailed evaluation of the various diagnostic modalities is outside the scope of this paper.

Investigation of such cases should also exclude the possibility of intrathoracic malignancy. HDP may also result from infiltration by tumors of the phrenic nerve at any level from the nerve roots to the lung and mediastinum, the most common being thymic and lung neoplasms [28]. Damage to the nerve may also result from treatment with radiotherapy or percutaneous ablation [29]. Kreisman et al. reported that four out of 39 cases of metastatic breast cancer resulted in phrenic nerve palsy [30]. In the two cases described here, the caring physicians did not order a CT scan of the thorax as the long-standing nature of the HDP practically excludes malignant neoplasia [30].

## Conclusions

This series of three cases shows that as both HDP and thoracic interventional medical/surgical procedures are not uncommon, they might coexist in the same patient. This means that the procedure may or may not cause paralysis. Investigating the patient's history should show a clear temporal relationship if the procedure is causal. If any episodes of dyspnea or chest pain occur postoperatively, damage to the phrenic nerve should be considered, along with other causes such as pulmonary embolus or lung collapse.

When the HDP is discovered months or years after a procedure on routine radiography of the chest, a thorough review of past radiography is necessary. When there is no temporal relationship with a procedure, primary or secondary intrathoracic malignancy must be excluded by means of CT thorax. The possibility of neurological disease, in particular cervical root compression at C3-C5 by disk prolapse, may also be considered and investigated with an MRI scan.
